# Lonely and connected in emerging adulthood: The ambivalence of sociality in a time of transitions

**DOI:** 10.1371/journal.pone.0334787

**Published:** 2025-11-12

**Authors:** Jeffrey A. Hall, Natalie Pennington, Amanda J. Holmstrom

**Affiliations:** 1 Department of Communication Studies, University of Kansas, Lawrence, Kansas, United States of America; 2 Department of Communication Studies, Colorado State University, Fort Collins, Colorado, United States of America; 3 Department of Communication, Michigan State University, East Lansing, Michigan, United States of America; University of Geneva: Universite de Geneve, SWITZERLAND

## Abstract

Utilizing a large, representative sample of Americans (*N* = 4,812), the present manuscript explores individuals’ age in relation to social well-being (i.e., connection, companionship, friendship support, and number of friends) and social ill-being (i.e., loneliness, disconnection). Participants ranged between the ages of 18–95, with a mean age of 43.7. Regression results demonstrate that although social ill-being is higher for emerging adults, social well-being is high for both younger and older adults. Cluster analysis identified four clusters of social ill-being and well-being. Participants in the cluster with high well-being and moderate ill-being were more likely to be young, educated females who had gone through many life changes in the past year. Participants in the cluster with high well-being and lowest ill-being were more likely to be older adults, facing fewer life changes. The present manuscript suggests that loneliness among young adults is not bereft of connection, companionship, and friendship, but instead is characteristic of rapid life changes and a lack of relational permanence and routine.

## Introduction

The U.S. Surgeon General’s report [1] on the effects of loneliness and isolation on health and well-being documents growing rates of loneliness in the U.S. Although high rates of loneliness among the oldest adults have been noted for some time [2,3], there is also evidence of a recent increase in loneliness among young adults [4–6], which has garnered national headlines and attention [7,8]. The Surgeon General’s report [1] stresses the importance of social connection as one path toward combatting loneliness. At any age, a lack of social opportunities or close relationships can be unhealthy [9], but the benefits associated with possessing social resources deserve research attention as well [10]. Indeed, human thriving includes not only the absence of ill-being but also the presence of well-being [11].

Using a large sample of Americans (*N* = 4,812), the present study explores the possibility that, relative to other age groups, emerging adults experience both high rates of social well-being *and* high rates of social ill-being. Drawing from conceptualizations of emerging adulthood [12] and ontological security [13], we identify the characteristics and attitudes of individuals who experience both positive and negative aspects of social health. The primary contribution of the present study is that it demonstrates that connection and disconnection can co-occur in the same group of people, particularly emerging adults. To understand the social health of young adults, this investigation adopts a more complete understanding of the unique challenges and transitions of young adulthood, marked by ontological insecurity and impermanence.

### Social ill-being and well-being

There is some disagreement regarding whether well-being is a bipolar or bivariate concept [10,14,15]. Although scholars seem to agree that “well-being is not deducible from the absence of ill-being” ([15], p. 650), well-being is negatively associated with ill-being both within- and between-individuals. Others have argued (e.g., [14]) that the presence of strong covariance does not diminish the benefits of measuring each concept separately. For example, there are distinct biomarker profiles for individuals who report high eudaemonic well-being and for those who report high depression and anxiety [10], reinforcing the value of bivariate, rather than bipolar, measures of social health. Similarly, research from Closson et al. [16] argues the need to explore positive aspects of well-being (e.g., life satisfaction, happiness) in relation to social interaction and the experience of solitude for emerging adults, not just negative outcomes. When well-being is broadly conceived as human flourishing, it includes aspects such as physical health, happiness, life satisfaction, purpose and meaning, and competence and achievement [11,17]. There is also agreement, however, that social aspects of well-being constitute a branch of flourishing that is distinct from emotional well-being, hedonistic pleasures, and competence.

Indeed, it is common for conceptualizations of eudaemonic well-being to include social connection, friendship, and social support [11,18]. When considering the degree to which social connection predicts health and longevity, Holt-Lunstad [9] argues that structural (e.g., social networks, social capital), functional (e.g., perceived support, loneliness), and quality (e.g., social inclusion or exclusion) factors are all relevant. Hawkley et al. [19] suggests that loneliness has three subfactors: intimate connectedness, relational connectedness, and collective connectedness. Other conceptualizations of loneliness separate intimacy and companionship [20]. It is beyond the scope of the present investigation to resolve the bipolar or bivariate nature of social well-being or to establish a comprehensive multifactorial measure of social health. In the present manuscript, measures of social well-being and ill-being were guided by developmental accounts of human sociality and the sociological concept of ontological security. In this investigation, we draw from past research on loneliness [9,19]. Social well-being was measured by companionship, connection, social support, and friendship network size, which integrates structural, functional, and quality sub-dimensions. In this investigation, social ill-being was measured by loneliness (e.g., intimacy) and social disconnection, drawing from functional and quality dimensions [19,21].

### Sociality throughout the lifespan

At all stages of life, close relationships, particularly friends and family, are important to well-being and happiness [5,6,22,23]. The role, function, and aspects of close relationships change throughout adult development [24–26]. A large meta-analysis of social network size and composition [27] suggests that close relationships are most bountiful between 15 and 25 years of age, but then social network size plateaus and declines in the late 20s. The number of friendships particularly drops steeply – one friend per decade thereafter. Similarly, a panel study of nearly 37 thousand German adults [28] suggests that social contact with friends peaks at 20 and then steeply declines until 45. Contact with friends levels out between 45–60 years of age before declining again from 60 to 80 years. Abundant friendships and social opportunities appear to be particularly predictive of well-being for young adults [29]. Although positive outcomes are associated with the quantity of social interactions for younger adults, interaction quality is more strongly associated with positive outcomes for older adults [3,29].

Scholars [24,28,29] have turned to a life history approach to explain these findings. Indeed, Vacchiano et al. [26] argue that it is important to take into consideration how different network members, such as acquaintances or colleagues, play a distinct role during different stages of one’s life. The primary developmental tasks of emerging adulthood are experimenting with one’s social and personal identity, which accompanies detachment from one’s family of origin and pursuit of new relationships [12]. Emerging adults focus on forming and growing relationships to practice relational commitment and to develop self-identity [12]. Friends are particularly valuable at this period as they are sources of companionship and support, often preceding the formation of committed romantic relationships [5,24,28].

Emerging adulthood is also marked by a series of tasks or accomplishments which precipitate dramatic changes in sociality. The acquisition of social capital through friendship reverses direction and declines when young adults form romantic partnerships, settle into careers, or have children [28]. These changes are quite common during emerging adulthood [12,30] and have practical consequences for social well-being. Beam and Kim [31] argue that social isolation and loneliness may be a larger problem for young adults, compared to middle-aged and older adults, because young adulthood is marked by dramatic change in – rather than stability of – social network composition, access to relational partners, and socio-emotional development. The sheer frequency of such changes between late adolescence and young adulthood may increase the likelihood of experiencing loneliness, particularly as routines are disrupted and family access and/or support may wane [31]. Moving away, changing jobs, starting and ending school, getting married, and having children are major life accomplishments that can precipitate the loss of friends [27,32]. Longitudinal studies suggest that a lack of opportunities to interact, particularly in person, is the biggest factor in losing a social network member over a seven-year window [33]. This was also seen during the COVID-19 pandemic; Völker [34] found that for younger participants (18–35), networks shrunk 10%, compared to roughly 5% for older participants (65 + years old).

At the close of emerging adulthood, a greater focus on romantic relationships and family accompanies a renewed focus on one’s family of origin. After years of abundant interactions with friends, around 35 years of age, friend contact is less frequent than family contact [28]. Building a new family often impedes friendship maintenance but mitigates loneliness [31,35]. Fortunately for adults, building workplace friendship accompanies middle adulthood [24,25]. Yet workplace relationships are unstable relationships [33]. Taking these factors into account, a life history perspective suggests people have often made most of the friends they are going to make in their lifetimes by early adulthood [27].

### Change and ontological security

The concept of ontological security provides a framework for understanding why emerging adulthood might be experienced as a duality – both connecting and isolating. Giddens [13,36] scrutinizes routine daily practices, as they reflect and constitute the individual’s understanding of the world and their place within it. Through daily rituals and behaviors, members of a society reconstitute the structure of society (i.e., the duality of structure) [37]. In all times of life, including emerging adulthood, new routines are built, which then reconstitute identity. Constant change, however, comes at a cost. When recursive practices are predictable – rendered routine – they create a sense of ontological security [13]. Giddens [38] writes, “business as usual” is “the prime element in the stabilising of trust and ontological security” (p. 492). “To be ontologically secure,” Giddens [13] states, “is to possess, on the level of the unconscious and practical consciousness, ‘answers’ to fundamental existential questions which all human life in some way addresses” (p. 47). Ontological security, however, requires an acceptance and a complete inhabitation of normative social structures. Life must become ordinary: predictable, stable, and routine.

In their analysis of loneliness over the lifespan, Luhmann and Hawkley [2] argue that different factors predict loneliness at different stages of life. Living alone and not working are more normative for young people, which may be why such indicators play a small role in the social health of young adults. By comparison, Luhmann and Hawkley found middle-aged and older adults’ social health was negatively affected by those factors. Similarly, social participation with friends and in the community predicts less loneliness for young adults. For middle-aged individuals, social time with close kin and romantic relationships predicts less loneliness [5]. For young adults, having many friends, social events to attend, and new people to meet, work with, and date all facilitate connection and companionship.

In the backdrop of this abundance, the very activities that are normative – starting and ending one’s education, changing jobs, initiating and ending romantic relationships, relocating [30] – forestall the ontological security that permanence and predictability could create [13,36]. Constant change precipitates loneliness as routines are disrupted [31]. Even important achievements, such as graduation from a degree program or starting a new job, require a disruption of a routine, leaving predictable sociality behind. The loss of routine allows the existential dilemmas that are hidden behind mundane daily practices to “present themselves in pressing form” ([13], p. 167). In Gidden’s terminology, ontological security is contingent on school, friendships, and new romantic relationships transitioning from initiation and experimentation to closure and permanence. This leaves many young adults feeling like they will become an adult once they get a ‘real’ job, once they find the ‘one’ to settle down with, and once they can afford an independent life. Rather than easily achieved, ontological security is perpetually deferred in the face of the constant change and anticipatory achievement that is the very definition of emerging adulthood.

Together, normative accounts of loneliness and ontological security suggest that the same factors that predict disconnection are the ones which are developmentally crucial during emerging adulthood. This supposition is well-supported; changes that are normatively positive (e.g., graduation, getting a job, forming a romantic partnership) are also socially disruptive, particularly to friendship [27,28,31]. The implications for social well-being are less well-understood. It stands to reason that abundant friends and social opportunities would increase social well-being. Strong friendships provide companionship in an uncertain world, with evidence friends are especially important when individuals have more ambivalent ties with their romantic partner [18]. Watters [39] argues that, for young adults, chosen families can be as important for social connection as traditional families. Compared to spouses, families of origin, and offspring, however, friendships are comparatively transitory and much less influential on regulating daily routines [13]. After all, independence is the very hallmark of friendship [40]. Young adults may perceive their friendship experience as both one of abundance and insecurity as they move from place to place and develop their careers and identities.

### The present investigation

In accord with past research, we assumed that social ill-being (e.g., loneliness, disconnection) is greater for younger and older adults as compared to middle-aged adults. Following from this reasoning, social well-being and age may also exhibit a curvilinear relationship, such that younger and older adults will exhibit lower well-being as compared to middle-aged adults. However, young adults have an abundance of opportunities to connect, compared to middle-aged and older adults, despite instability in their social networks. These opportunities to connect would suggest a negative association between social well-being and age. In the present investigation, we explore both the linear and curvilinear associations between age and social well-being and age and social ill-being:

RQ1: How do social well-being and social ill-being vary with age?

The present investigation will also examine the co-occurrence of social well-being and ill-being. Conceptually, four different combinations of social health are possible. The first two combinations represent congruence in measures of social health, with the first representing overall good social health (i.e., high well-being and low ill-being) and the second representing overall poor social health (i.e., low well-being and high ill-being). The second two combinations represent social ambivalence: high well-being and high ill-being; low well-being and low ill-being. We will examine the characteristics of individuals who experience each of these four quadrants:

RQ2: What are the demographic, social circumstances (i.e., living, dating), life changes, and attitudes about relationships of those who are in each of the four quadrants of social health?

## Materials and methods

### Participant recruitment

In July and August of 2022 and June and July of 2023, two quota samples of American adults were recruited by the Siena College Research Institute (SCRI), a national leader in public opinion polling. Adult participants were provided an IRB-approved information statement and written consent on an online form. IRB approval for the 2022 survey was obtained for the project by the University of Kansas Human Subjects Committee Lawrence (IRB #STUDY00147041). The 2023 survey IRB approval was obtained from the University of Nevada, Las Vegas (IRB #UNLV-2022–423) and Michigan State University (IRB #STUDY00008066). In 2022 (*N* = 2,034) and in 2023 (**N* *= 2,243) participants completed demographic measures, then completed a name generator task, answered questions about friendship generally, and finally answered questions about their social health (i.e., social well-being, social ill-being). Several attention check and quality control procedures were used to ensure data quality, and all participants responded to all survey measures.

In September through December of 2022 and 2023 to match the portion of student participants in the U.S. population particularly among young adults, the co-project leaders fielded the same survey at their respective universities located in the Midwest, the upper Midwest, the Southeast, and the West. International student participants were excluded. Adult participants that were recruited from universities (called “student participants” hereafter) were provided an IRB-approved information statement and a written consent on an online form.

All participants in the quota sample who indicated they were over the age of 22 years and were students were included in the present dataset (2022: *n* = 138; 2023: *n* = 83). Then, a random sample of 18–21-year-old student participants with complete answers to all survey questions were included in the present dataset from each year (*n* = 151). A total of 562 student participants were added to the dataset. The portion of students among young adults in this sample (42.5% of 18–24 y.o.) approximates the proportion of students among young adults in the U.S. overall (38.5%) [41].

### Participant demographics

The final sample included 4,812 adult participants. Compared to U.S. census data [42], 22% of the sample was 18–25 (compared with 13.5% of U.S. adult population), 15% of the sample was 26–34 (compared with 15.7% of U.S. adult population), 34% of the sample was 35–54 (compared to 33% of U.S. adult population), 14% of the sample was 55–64 (compared to 17% of U.S. adult population), and 15% of the sample was 65+ (compared to 22% of U.S. adult population). The final sample overrepresented young adults and underrepresented older adults. However, as the focus of this investigation was on emerging adults, their greater proportion in the sample helped to capture variability in young adult experiences.

Participants ranged between the ages of 18–95, with a mean age of 43.7 (*SD* = 18.0). Participants were most likely to identify as female (57.4%) or male (41.7%), with.9% trans-identified, non-binary, or other gender. Participants primarily identified as heterosexual (87.7%), with 7% of respondents identifying as bisexual, gay (1.7%), lesbian (1.3%), and other self-identified sexualities (2.3%). Most participants identified as not Hispanic/Latino/a/x (88.9%), 11.1% identified as Hispanic/Latino/a/x. Participants were allowed to identify as many races as they wished, and 3.3% identified as multi-racial. Of those identifying one race, the majority identified as White (76.4%), Black or African American (11.7%), Asian (4.0%), some other race (3.0%), or American Indian or Alaska Native or Native Hawaiian (1.5%).

Participants were primarily single (30.8%), married or cohabitating (44.0%), or dating (9.4%), and 15.8% indicated they were divorced, separated, widowed, or in an “other” type of romantic relationship. Most participants (57.8%) were employed, of which 71.1% were working full-time. Participants indicated their highest level of education on an 8-point ordinal scale [mode = completed high school (36.1%), median = associate's degree (currently enrolled)].

### Social health

The two measures of social ill-being were loneliness and social disconnection. *Loneliness* [43] included three items, and was rated on a three-point scale (hardly ever-often) (*M* = 1.76, *SD* = .67, α = .86). Hughes et al. [43] reports on the development and validation of this scale, which is a shortened adaptation of the Revised UCLA Loneliness Scale. According to Hawkley and colleagues’ analysis [19], the Hughes et al. [43] scale only includes items measuring the absence of intimate connection. *Social disconnection,* consisting of four items from Lok and Dunn’s [21] disconnection sub-scale, was rated on 7-point Likert-type scales ranging from 1 (strongly disagree) to 7 (strongly agree) (*M* = 3.72, *SD* = 1.71, α = .92). The four measures of social well-being were social connection, companionship, perceived support, and number of friends. *Social* c*onnection* [21] (six items) was rated on 7-point Likert-type scales (*M* = 5.00, *SD* = 1.25, α = .90). The measure of c*ompanionship* [44] consisted of six items rated on 5-point Likert-type scales ranging from 1 (never) to 5 (always) (*M* = 3.77, *SD* = 1.02, α = .90). *Perceived social support* [45] was assessed with four items rated on a 7-point Likert type scale (*M* = 5.80, *SD* = 1.09, α = .88). Finally, *number of friends* was measured by a name generation task. Participants could list up to six (2023) or seven (2022) names or initials of individuals who they considered friends. Participants answered questions about each of the names they listed. Most participants identified at least one friend (98%) (*M* = 4.36, *SD* = 2.00). Appendix A reports all items for all scales.

Ontological security was operationalized in several ways. First, participants answered yes or no to whether they had experienced any of 13 *life changes* in the past year (*M* = 1.53, *SD* = 1.06). Changes included: started school; gotten married; started a new job; stopped working/retired; moved to a new place; adopted a child or had kids; had (step) children move out of the house; experienced the death of a romantic partner; experienced the death of a household member; moved to a new city, state, or country; started a new romantic relationship; ended a romantic relationship (including separation and divorce); and graduated from school or degree program. Second, participants indicated if they had in the last year *lost touch with any of your friends* (39.6% indicated “yes”). Third, participants also reported their *perceived stress* [46] by responding to four items rated on 5-point Likert type scales ranging from 1 (never) to 5 (always) (*M* = 2.72, *SD* = .84, α = .80). See Table 1 for correlation matrix.

**Table 1:** Correlation matrix.

	1	2	3	4	5	6	7	8
1 Loneliness								
2 Disconnection	.757^*^							
3 Connection	−.537^*^	−.633^*^						
4 Companionship	−.547^*^	−.505^*^	.587^*^					
5 Perceived support	−.310^*^	−.358^*^	.499^*^	.407^*^				
6 Number of friends	−.092^*^	−.148^*^	.252^*^	.215^*^	.166^*^			
7 Life changes	.156^*^	.135^*^	0.009	.045	0.035	.164^*^		
8 Lost touch w/ friend	.282^*^	.268^*^	−.113^*^	−.067^*^	−.108^*^	.128^*^	.259^*^	
9 Perceived stress	.627^*^	.659^*^	−.547^*^	−.458^*^	−.261^*^	−.060^*^	.139^*^	.231^*^

*Note*: * **p* *< .001; *N* = 4,812

If participants had at least one friend listed in the name generation task, they answered six single-item questions regarding their attitudes and experiences in close relationships and friendships using a 7-point Likert type scale ranging from 1 (strongly disagree) to 7 (strongly agree): “In the past year, I am satisfied with how well I have maintained friendships,” “I am satisfied with the number friends I have,” “I am satisfied with the amount of time I am able to spend with my friends,” “My friends celebrate my good news,” “It is difficult to make new friends,” and “In the past year, maintaining close relationships has been difficult and frustrating for me”.

To test the first RQ, six OLS regressions were run with linear and quadradic terms for age. To answer RQ2, cluster analysis was used to establish groups of participants and then to profile members of each quadrant. Then, a polynomial logistic regression was conducted to explore which characteristics of individuals predicted being a member of each cluster. All analyses were performed in SPSS version 29.

## Results

**Fig 1:**
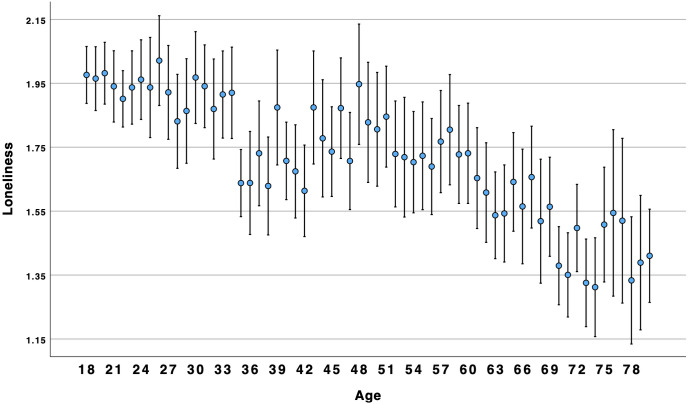
Age by loneliness.

**Fig 2:**
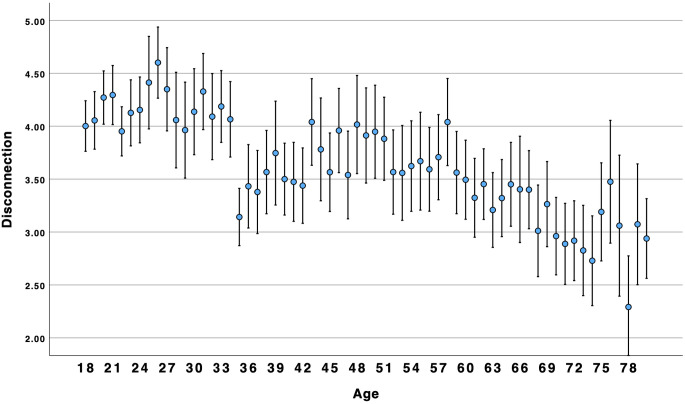
Age by disconnection.

To test RQ1, the first two regressions explored the linear and quadradic association between age and social ill-being. No covariates were included in these analyses. The first regression demonstrated that age showed a significant, negative linear relationship with loneliness, age: unstandardized *b* = −.008, *SE* = .001, *p* < .001, standardized *b* = −.226; *R*^2^ = .05. The second regression analysis demonstrated that age showed a significant, negative linear relationship with disconnection: unstandardized *b* = −.019, *SE* = .001, *p* < .001, standardized *b* = −.200; *R*^2^ = .04. Curvilinear terms were estimated, but neither were significant: loneliness, age^2^ *p* = .091; disconnection, age^2^ *p* = .196. In response to RQ1, the relationship between age and social ill-being was linear. Figs 1 and 2 illustrate the downward trend in loneliness and disconnection from young adulthood to middle-age to older adulthood. In all figures, participants from 78–95 were combined into a single age category for illustration purposes.

To further answer RQ1, four regressions explored the linear and quadradic associations between age and social well-being. No covariates were included in these analyses. The first regression demonstrated that age showed a curvilinear relationship with connection, age: unstandardized *b* = −.032, *SE* = .006, *p* < .001, standardized *b* = −.461; age^2^: unstandardized *b* = .000, *SE* = .000, *p* < .001, standardized *b* = .482, *R*^2^ = .01. The second regression analysis demonstrated that age showed a curvilinear relationship with companionship: unstandardized *b* = −.029, *SE* = .005, *p* < .001, standardized *b* = −.509; age^2^ unstandardized *b* = .000, *SE* = .000, *p* < .001, standardized *b* = .460, *R*^2^ = .01. The third regression analysis demonstrated that age showed a curvilinear relationship with number of friends: unstandardized *b* = −.117, *SE* = .009, *p* < .001, standardized *b* = −1.052; age^2^ unstandardized *b* = .001, *SE* = .000, *p* < .001, standardized *b* = .962, *R*^2^ = .04. The fourth regression analysis demonstrated that age showed a linear relationship with support: unstandardized *b* = −.004, *SE* = .001, *p* < .001, standardized *b* = −.64; *R*^2^ = .004. The quadradic term was not significant: age^2^ unstandardized *b* = .000, *SE* = .000, *p* = .287.

**Fig 3:**
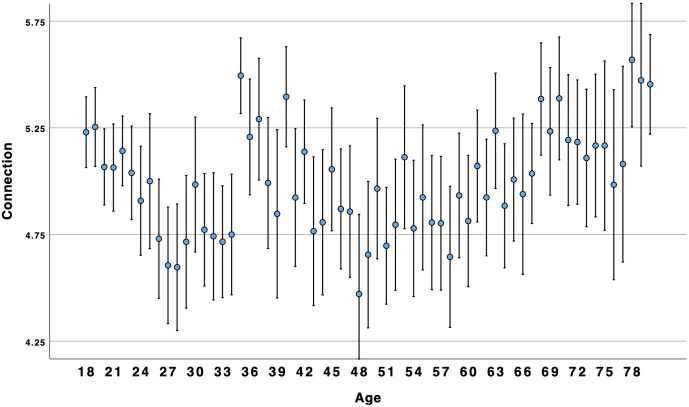
Age by connection.

**Fig 4:**
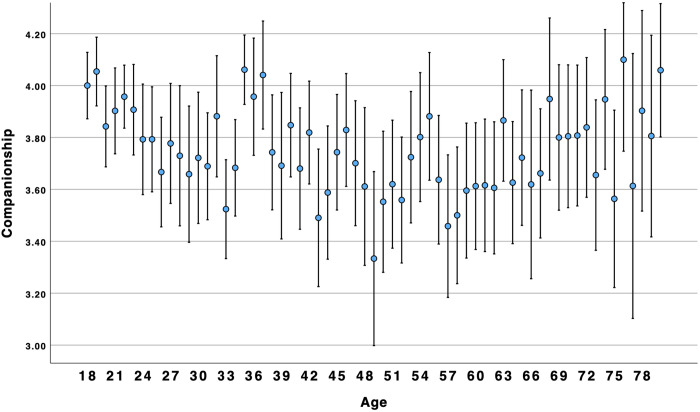
Age by companionship.

**Fig 5:**
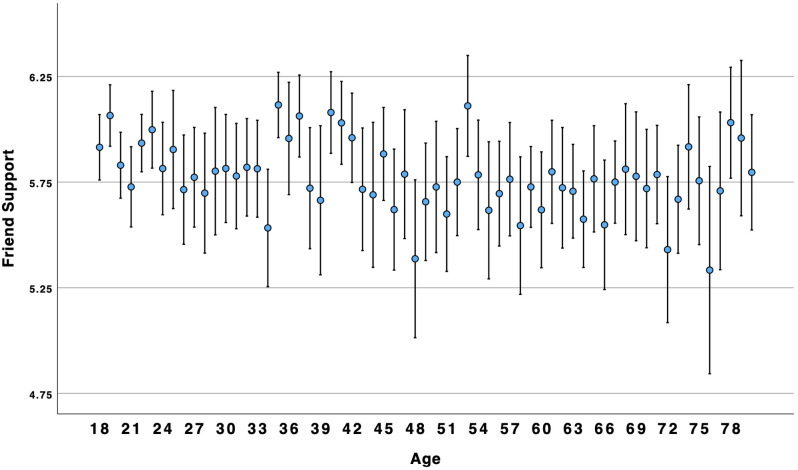
Age by friendship support.

**Fig 6:**
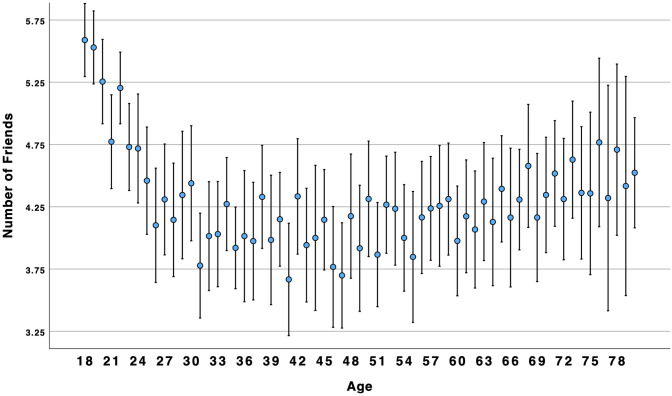
Age by number of friends.

There was no support for the supposition that connection, companionship, and number of friends would show a similar pattern as loneliness and disconnection. Results for friendship support supported a linear association. Although there was a significant curvilinear association for the other three variables, it was in the opposite direction as expected. Overall, the results suggested that connection, companionship, and number of friends, were similar to social ill-being: higher for young adults and older adults alike (see Figs 3–6).

### Cluster analyses

Cluster analysis is used to identify distinct profiles of participants based on scores for a set of theoretically relevant variables, which were the six measures of social ill-being and well-being. Theoretically, four classifications were presumed: high well-being/high ill-being; high well-being/low ill-being; low well-being/low ill-being; low well-being/high ill-being. All well-being and ill-being measures were used to profile participants falling in each of the quadrants. K-means cluster analysis was used to specify a four-cluster solution (Table 2). Even when categories are theoretically expected, cluster analysis forms groups through patterns in the data, which does not always yield the exact groups hypothesized. Given the normal distribution, cluster analysis frequently identifies groups that are moderate across several variables. Cluster one (*n* = 2,944) included 61% of participants, and was characterized by high social well-being (companionship, connection, number of friends, friendship support) and moderate levels of ill-being (disconnection, loneliness). Participants in cluster one were ambivalent about their social health. Cluster two participants (*n* = 798) were high in social well-being and low in social ill-being, albeit they had fewer friends than all other clusters except one. Cluster two was the high social health cluster (i.e., high well-being/low ill-being). Cluster three participants (**n* *= 915) reported the fewest friends, and moderate levels of both social ill-being and well-being – representing a moderate social well-being group. Cluster four participants (*n* = 155) were the lowest in social well-being and highest in ill-being, representing poor social health.

**Table 2:** Cluster Analysis Results.

	Cluster 1	Cluster 2	Cluster 3	Cluster 4
	Ambivalent	Social Well-Being	Moderates	Social Ill-Being
*N*	2,944	798	915	155
	*M*	*M*	*M*	*M*
Loneliness	1.69	1.27	2.27	2.62
Disconnection	3.61	1.98	5.21	5.87
Connection	5.50	5.04	3.74	2.95
Companionship	4.04^a^	4.07^a^	2.85	2.46
Number of friends	5.23^a^	3.90	1.84	5.18^a^
Friend support	6.05	5.93	5.22	3.38

*Note*: For all variables, mean values are significantly different at *p* < .001 from every other cluster, except when they share “a”

### Multinominal logistic analyses

To answer RQ1, multinominal logistic analyses were conducted, with the ambivalent group (cluster 1) set as the referent group. The first analyses included all participants (Table 3, columns 1, 3, 5). The second analyses included only individuals who reporting having at least one friend (Table 3, columns 2, 4, 6). Members of the ambivalent group were younger and had more education than individuals with high or moderate social well-being. They were also more likely to have experienced more life changes than other clusters. Other characteristics that distinguished the ambivalent group were idiosyncratic. For example, ambivalent group members were more likely to be White, female, and dating when compared to the moderate well-being group members, who were more likely Black, male, and married. Stress also showed a distinct pattern by group, where the high social well-being members were less stressed than the ambivalent group members, but the ambivalent group members were less stressed than moderate and low social well-being group members. Furthermore, high social well-being group members were less likely to have lost touch with a friend than ambivalent group members, while high social ill-being group members were more likely to have lost touch with a friend than ambivalent group members.

**Table 3:** Unstandardized estimates for the logistic regression of participant characteristics and attitudes predicting cluster membership.

	Cluster 1 Cluster 1		Cluster 2 Cluster 2		Cluster 3 Cluster 3		Cluster 4 Cluster 4	
All Participants								
Age	−.011 (.002)**	−.011 (.003)**	.011 (.003)**	.013 (.003)**	.006 (.003)	.008 (.003)	.004 (.007)	.0012 (.007)
Male	−.224 (.065)**	−.116 (.069)	−.023 (.085)	−.040 (.088)	.334 (.084)**	.256 (.092)*	.033 (.185)	−.097 (.204)
Trans-, Non-.	.193 (.341)	.198 (.357)	−.607 (.744)	−1.215 (.999)	−.201 (.418)	−.195 (.427)	.160 (.632)	−.077 (.752)
Latinx = 1	−.097 (.108)	−.113 (.300)	.221 (.146)	.251 (.149)	.113 (.137)	.194 (.147)	−.450 (.335)	−.485 (.353)
Black	−.275 (.098)*	−.360 (.104)**	.168 (.132)	.146 (.136)	.510 (.119)**	.692 (.131)**	−.910 (.358)*	−.579 (.388)
Asian	−.193 (.161)	−.183 (.166)	.436 (.202)	.387 (.206)	.209 (.213)	.297 (.229)	1.838 (1.02)	−1.416 (.999)
Native Ame.	−.364 (.275)	−.113 (.300)	−.183 (.400)	−.166 (.414)	.682 (.314)	.358 (.359)	−.184 (.746)	.194 (.776)
Multiracial	−.062 (.176)	−.148 (.184)	.174 (.238)	.209 (.213)	.046 (.227)	.114 (.242)	−.342 (.476)	−.161 (.516)
Other race	−.205 (.186)	−.163 (.198)	−.298 (.285)	−.228 (.290)	.572 (.217)*	.537 (.231)	−.610 (.631)	−.808 (.660)
Education	.105 (.016)**	.091 (.017)**	−.060 (.021)*	−.046 (.021)	−.093 (.021)**	−.089 (.023)**	−.119 (.049)*	−.126 (.054)*
Employed = 1	.017 (.070)	−.099 (.072)	.264 (.096)**	.250 (.099)*	−.206 (.088)	−.054 (.096)	−.073 (.185)	.098 (.202)
Dating	.502 (.133)**	.503 (.138)**	−.353 (.196)	−.308 (.199)	−.537 (.179)*	−.583 (.190)*	.253 (.317)	.244 (.339)
Single	−.024 (.087)	.038 (.093)	−.175 (.121)	−.165 (.123)	.235 (.110)	.153 (.119)	.087 (.244)	.066 (.272)
Div/Wid/Sep	.060 (.100)	.075 (.105)	−.331 (.135)*	−.323 (.138)*	.213 (.128)	.207 (.136)	.285 (.264)	.295 (.240)
Live alone = 1	−.035 (.084)	.015 (.089)	−.163 (.120)	−.185 (.123)	.190 (.105)	.179 (.114)	.235 (.218)	.295 (.240)
# Life Change	.172 (.035)**	.147 (.037)**	−.180 (.057)*	−.169 (.058)*	−.132 (.045)*	−.114 (.047)*	−.063 (.085)	−.020 (.088)
Stress	−.474 (.041)**	−.295 (.048)*	−.710 (.057)**	−.465 (.064)**	1.106 (.055)**	.830 (.065)**	1.149(.105)**	.536 (.129)**
Lost friend = 1	.202 (.067)*	.225 (.072)*	−.431 (.096)**	−.279 (.099)*	−.081 (.085)	−.161 (.094)	.752 (.183)**	.246 (.203)
At least 1 friend								
Satis. maintain friend		.222 (.030)**		−.112 (.043)*		−.125 (.037)**		−.305 (.080)**
Satis. # of friends		.091 (.026)**		.086 (.038)		−.133 (.031)**		−.116 (.060)
Satis. time w/ friends		−.087 (.023)**		.070 (.031)		.042 (.030)		−.087 (.066)
Friend celebrate news		.315 (.031)**		−.131 (.041)**		−.216 (.037)**		−.620 (.067)**
Diff. make friends		−.083 (.020)**		−.030 (.025)		.210 (.028)**		.171 (.067)*
Frustrating maintain close relationships		.146 (.026)**		−.248 (.035)**		.007 (.034)		.067 (.084)
Goodness-of-fit								
Cox & Snell	.069	.115	.080	.102	.128	.158	.044	.088
*N* participant	4,218	4,711	4,812	4,711	4,812	4,711	4,812	4,711

*Notes*: *p* < .001; Reference group for alter (Level 1) is female, living outside of house, and member of discussion network. Reference group for participant (Level 2) is female, non-Latinx, white, currently not employed, single (not dating), and live with other people

For participants who had at least one friend, ambivalent group members were more satisfied with how well they’d maintained close relationships in the past year and more likely to have friends who celebrated their good news compared with all other groups. Compared to two other groups, they were more satisfied with the number of friends and were also less likely to say it was difficult to make new friends. Compared to the high social well-being group specifically, ambivalent group members were less satisfied with time spent with friends, and more likely to say they were frustrated with how well they’d maintained close relationships.

## Discussion

Consistent with recent public reports [7,8] and past research [31], the present investigation demonstrated that emerging adulthood is characterized by loneliness and disconnection. Social ill-being is higher for emerging adults than for any other age group. Despite these experiences of social ill-being, emerging adults also experienced high social well-being. Unlike older adults, who showed a congruent combination of social health (i.e., low in loneliness, high in connection), young adults were typically ambivalent in their social experiences. Cluster analysis demonstrated that this ambivalence was characterized by high relational turnover, dating, and many life changes, but less overall perceived stress. By contrast, those who had the highest social ill-being were less educated and more stressed. This suggests there are important differences in the experience of loneliness among emerging adults and the experiences of socially disconnected adults.

### Social well-being over the lifespan

Concerns about high and growing rates of loneliness among young adults have been reported for many years [2] and throughout the world [4]. Indeed, our results suggest that younger Americans are more likely than older adults to experience social ill-being (i.e., loneliness, disconnection). However, we found that social well-being (i.e., connection, companionship, friendship support, number of friends) was higher among younger and older adults as compared to middle-aged Americans. Results suggest that focusing on negative aspects of sociality, such as loneliness, may obscure the positive aspects of sociality, such as connection. This suggests that these two are not opposites; rather, akin to attitudes and global well-being [10,14], social well-being can be experienced in both positive and negative ways within the same person. This is consistent with Closson et al.’s [16] argument that it is important to account for both positive and negative outcomes from social interaction. Figs 3 and 4 illustrate a curvilinear pattern for these positive aspects: connection and companionship are the highest for young adults, decline in middle age, and then rise again for older adults. A decline in connection and companionship after 30 years of age is consistent with longitudinal research on friendship [47]. However, Figs 1 and 2 illustrate a linear pattern for ill-being – loneliness and disconnection decline from younger to middle-aged adults and from middle-aged to older-adults. Young adults experience an intense *ambivalence* about their social health – the structure and quality of their relationships is high, but they feel more disconnected and lonelier than other age groups.

Cluster analysis added nuance to these findings. We found that high social well-being and moderate social ill-being were characteristic of young and educated individuals who had experienced more life disruptions. These trends fit into broader discussions about life history perspectives [28,35] and normative explanations of loneliness [2]. The churn of friendships and romantic relationships from high school to college through the 20s is highly characteristic of that stage of life [12,31], as individuals experience more changes in life (e.g., moving for college, moving for a job). The attitudes toward friendship also reflected their ambivalence. They were more likely satisfied with the number of friends they had and how well they had maintained close relationships, and they had friends who celebrated good news. They also felt that it wasn’t difficult to make new friends. Yet, they were dissatisfied with the amount of time they spent with friends, when compared to those with the highest social well-being.

One explanation for these results could be that emerging adults strive to meet a high threshold of connection. They may find more difficulty with shifting expectations and time demands as they age, struggling with how to balance and spend time with established connections they care about and demands with work and family. These individuals are happy with their connection and companionship in a broad sense, but have difficulty making time for relationships, resulting in that tension between high well-being and high ill-being. Furthermore, compared to individuals with moderate social well-being *and* ill-being, ambivalent individuals were more likely to be women. More than men, women tend to have higher expectations of friendship, both in terms of closeness and communication, and tend to take on more responsibility for friendship upkeep [48]. This results in more intimate friendships but also a greater risk of disappointment with friends, if friends are unable to meet high expectations.

Participants with the highest social well-being and lowest ill-being reported considerable routine and predictability. They were older and less likely to experience life changes, and less stressed. These particular older participants appear to enjoy a satisfying degree of predictability and stability in their lives – what Giddens [38] calls “business as usual.” Their attitudes suggested they were satisfied with their time with friends and felt maintenance of friendship wasn’t particularly difficult or frustrating although they, as a group, had fewer friends than those in the ambivalent cluster. They were less likely to have lost touch with a friend, despite having fewer friends than ambivalent group members.

By comparison, those in cluster three, which included people who were moderate in both social well-being and ill-being, also reported less education and more stress than the ambivalent group. They were also less likely to experience life changes or events. Participants were less likely to have friends to celebrate their good news and less satisfied with the number of friends they had, which reflected their low number of friends overall (*M* = 1.84). They were more likely than the ambivalent group to express that it was difficult to make new friends. Past research partially supports these findings, with evidence that education level was related to the size of one’s social network [32].

Finally, the group with the highest social ill-being and lowest social well-being (i.e., cluster four) were less educated and highly stressed. These results confirm past investigations of loneliness, including that a lack of education [49] and stress [50] are predictive of higher loneliness. They lacked friends to celebrate good news, were unsatisfied with maintaining close friends, and reported it was difficult to make new friends, and were more likely to have lost touch with a friend. Taken together, the results demonstrate that individuals who are the least socially healthy have a qualitatively different experience than those whose social well-being is ambivalent.

### Implications for ontological security

Although it was the case that young, educated individuals with many life changes were more likely to experience this social health ambivalence, nearly two-thirds of the entire sample was grouped into the ambivalent cluster. The predominance of this cluster type suggests that, like well-being in general [10] the two components of sociality (i.e., well-being, ill-being) are not diametrically opposed. Although these changing life events are characteristic of emerging adulthood, it is consistent with the idea that ontological security is challenged when life disrupts routines, which can happen at any age. The typical response to disruption is disorientation, uncertainty, and a search for new points of reference to attain ontological security [13]. Furthermore, it is important to point out that many of the disruptions that predicted being in the high social well-being/moderate social ill-being cluster were changes typically viewed as positive: marriage, having children, starting a new job, graduation. These changes can powerfully reorient individuals and eventually establish certainty, once routine is established [13]. The present investigation suggests that social well-being is often present as individuals adapt to changes – both good and bad. Indeed, these participants reported less stress despite a great deal of change in their lives.

By contrast, individuals with the least disruptions – the most stasis – were the most socially healthy. This, however, does not imply that a *lack* of changes predicts social well-being. Although individuals with the highest social health experienced less change, individuals in cluster three, who experience moderate social ill-being and moderate social well-being, also experienced few life changes. Indeed, individuals in both groups expressed dissatisfaction with how well they had maintained close relationships and were less likely to have friends to celebrate their good news. Perhaps the key difference for those who experience fewer life changes was the presence of stress, which was less likely for those with high social well-being and more likely for those with moderate well-being.

### Limitations

The present study is not without limitations. The cross-sectional survey design means that demographic characteristics, life changes, and attitudes cannot be presumed to cause social well-being or ill-being. Furthermore, the sample attempted to be representative of the U.S. population, but it lacked the oldest of adults. Losses of family, friends, and spouses that explain spikes in loneliness for the oldest adults [2], and their experiences were not captured in the present investigation. Furthermore, the findings point to variability in terms of demographics, particularly in the cluster analyses. This suggests additional analysis and theory is needed to better understand differences between racial groups on experiences of connection and disconnection.

It is also possible some of the ambivalence detected is an enduring consequence of the COVID-19 pandemic. The pandemic’s social distancing requirements may have had a particularly strong impact on emerging adults, who may have been developmentally more susceptible to these losses as these are crucial tasks associated with this developmental stage [12,28,34]. The size of the first cluster (61%) suggests that although young, educated individuals were more likely to have this experience, the pandemic could have left many Americans with enduring ambivalence about their social health.

Finally, because this investigation is unusual in that it explores both social ill-being and social well-being in the same study, it cannot be aligned with prior research. It was also not possible to compare to prior cohorts, so it is possible high rates of social well-being are a contemporary rather than historical phenomenon. Furthermore, although this manuscript relies upon the idea that social health includes both positive and negative dimensions, it is neither exhaustive nor conclusive in its constituent parts. In other words, future work may seek to document what are the primary aspects of both high and low social health.

## Conclusions and future directions

The most important implication of the present investigation is that loneliness and disconnection among young adults do not occur in the absence of social well-being. Many supportive, satisfying friendships and many meaningful life changes and transitions are characteristic of this time. Though evidence that young adults are at risk of loneliness was found in the present study as in others, the present investigation offers a more nuanced view about their social well-being. An alternative interpretation of the data draws from research that suggests loneliness includes both an intimacy component and a companionship component [19,20,35]. Intimacy is more strongly associated with an individual’s relationship with one person, rather than the frequency of interactions or size of their social network [19,35]. If life changes and transitions are more disruptive or preventative of intimacy with a romantic partner, best friend, or roommate, it could be that young adults both feel they have companions but still long for the intimacy of an established relationship. Past research from Karney [51] highlighted how attending college typically delays marriage and having children for women, framing socio-economic status as a buffer to external stress that allows the opportunity to support well-being. As the present study shows, however, this is a double-edged sword, with college-educated women representing those who are the most connected but moderately disconnected (i.e., the ambivalent group), likely due to prolonged periods of change.

The second contribution of the present investigation is developing the concept of ontological security in relation to loneliness among young adults. It is possible that the ‘accomplishments’ of emerging adulthood are becoming further forestalled in our contemporary economic and social climate. Thirty years ago, Rindfuss [30] pointed to demographic changes to argue that the accepted norms of young adulthood – school, work, marriage, and children – were becoming less normative. By the advent of the 21st century, a predictable path of young adulthood (e.g., marriage, children, employment) was further delayed or deferred into the future [39]. An increasing variety of lifestyle choices and pathways open to young Americans are now more normative than the traditional linear track to marriage and family. For example, recent U.S. census data reports a growing number of single adult households [52], and a report from the Pew Research Center noted that the majority of single adults surveyed were not seeking to start a romantic relationship [53]. To the degree that contemporary options open new possibilities for education, career experimentation, personal exploration, and disassembling heteronormative or sexist norms, they should be celebrated. The concept of ontological security suggests, however, that more options, more change, and a longer period of deferred stability, might bring about higher rates of loneliness and disconnection and a great deal of variability in social health [31]. If the period of time that young people negotiate uncertainty before accomplishing the traditional ‘stable’ life is becoming longer and longer, then it is conceivable that rates of loneliness and disconnection will rise as a greater portion of individuals are waiting a longer period of time for stability to arrive.
